# Mouse Leydig Cells with Different Androgen Production Potential Are Resistant to Estrogenic Stimuli but Responsive to Bisphenol A Which Attenuates Testosterone Metabolism

**DOI:** 10.1371/journal.pone.0071722

**Published:** 2013-08-15

**Authors:** Iuliia Savchuk, Olle Söder, Konstantin Svechnikov

**Affiliations:** Pediatric Endocrinology Unit, Department of Women’s and Children’s Health, Karolinska Institutet, Stockholm, Sweden; Bascom Palmer Eye Institute, University of Miami School of Medicine, United States of America

## Abstract

It is well known that estrogens and estrogen-like endocrine disruptors can suppress steroidogenic gene expression, attenuate androgen production and decrease differentiation of adult Leydig cell lineage. However, there is no information about the possible link between the potency of Leydig cells to produce androgens and their sensitivity to estrogenic stimuli. Thus, the present study explored the relationship between androgen production potential of Leydig cells and their responsiveness to estrogenic compounds. To investigate this relationship we selected mouse genotypes contrasting in sex hormone levels and differing in testosterone/estradiol (T/E_2_) ratio. We found that two mouse genotypes, CBA/Lac and C57BL/6j have the highest and the lowest serum T/E_2_ ratio associated with increased serum LH level in C57BL/6j compared to CBA/Lac. Analysis of steroidogenic gene expression demonstrated significant upregulation of Cyp19 gene expression but coordinated suppression of LHR, StAR, 3βHSDI and Cyp17a1 in Leydig cells from C57BL/6j that was associated with attenuated androgen production in basal and hCG-stimulated conditions compared to CBA/Lac mice. These genotype-dependent differences in steroidogenesis were not linked to changes in the expression of estrogen receptors ERα and Gpr30, while ERβ expression was attenuated in Leydig cells from C57BL/6j compared to CBA/Lac. No effects of estrogenic agonists on steroidogenesis in Leydig cells from both genotypes were found. In contrast, xenoestrogen bisphenol A significantly potentiated hCG-activated androgen production by Leydig cells from C57BL/6j and CBA/Lac mice by suppressing conversion of testosterone into corresponding metabolite 5α-androstane-3α,17β-diol. All together our data indicate that developing mouse Leydig cells with different androgen production potential are resistant to estrogenic stimuli, while xenoestrogen BPA facilitates hCG-induced steroidogenesis in mouse Leydig cells via attenuation of testosterone metabolism. This cellular event can cause premature maturation of Leydig cells that may create abnormal intratesticular paracrine milieu and disturb proper development of germ cells.

## Introduction

Growing number of evidence indicate that estrogens can play an important role in the regulation of Leydig cell function and steroidogenesis at different stages of their development. Previous studies have shown that 17β-estradiol (E_2_) preferably suppresses the expression and function of cytochrome CYP17 in vivo and in vitro [1], [2] and has potential to attenuate the regeneration of adult Leydig cell populations in the ethane dimethylsulfonate (EDC)-treated adult rats [3]. However, these earlier studies did not identify the type of estrogen receptors (ERs) responsible for the observed negative effects of estrogens on testicular steroidogenesis. Investigation of the role of estrogens in the regulation of testicular steroidogenesis by using genetically modified mice has demonstrated that ERα null (αERKO) mice had higher levels of serum testosterone and luteinizing hormone (LH) associated with activated steroidogenesis in their Leydig cells compared to wild-type mice [4]. These findings suggested that enhanced capacity for androgen biosynthesis by Leydig cells from αERKO mice was mediated by elevated levels of LH observed in these mice [4], but whether ERα plays a role in direct regulation of Leydig cell steroidogenesis was still unclear. Further studies with mice lacking functional ERα or ERβ provided clear evidence that estrogen-dependent attenuation of steroidogenic gene expression in fetal Leydig cells is mediated by ERα-dependent signaling [5], [6]. Similarly, using human aromatase-expressing transgenic male mice (AROM^+^) crossed with αERKO mice, Strauss and co-workers [7] have reported that the structural and functional disorders in Leydig cells caused by estrogen exposure were mediated via the ERα. Finally, recent study has shown that ERα-mediated signaling attenuated the expression and function of Nur77, a transcription factor that regulates the expression of several steroidogenic genes [8].

Growing number of evidence indicate that environmental chemicals with estrogenic activity can also bind to ERα, mimic action of E_2_ and suppressing androgen production in Leydig cells at different stages of their development [9]–[11] and thereby negatively effect on the proper formation of reproductive organs and reproductive potential. One of such estrogen-like compounds widely used in industry is bisphenol A (BPA). This xenoestrogen is able to bind and activate both ERα and ERβ [12] and is shown to suppress androgen production by rat Leydig cells in vivo and in vitro [9]. However, direct effects of BPA on androgen production by developing mouse Leydig cells with different capacity to produce androgens have not been investigated yet. Prepubertal Leydig cells produce androgens that play an important role in the initiation and maintenance of spermatogenesis as well as the regulation of multiple androgen-dependent physiological processes in the developing body [13].

Thus, since ERα-mediated signaling causes suppressive effects on steroidogenesis in mouse Leydig cells, the aim of the present study was to explore the relationship between androgen production potential of Leydig cells, their expression profile of estrogen receptors (ERs) and responsiveness to ERs agonists and estrogen-like endocrine disruptor BPA in selected mouse strains.

## Materials and Methods

### Materials

Dulbecco’s Modified Eagle’s Medium (DMEM) - Ham’s nutrient mixture F-12, Modified Eagle’s Medium (MEM), Hank’s Balanced Salts Solution (HBSS) without Ca^2+^ or Mg^2+^ and penicillin-streptomycin were all obtained from Gibco/BRL (Life Technologies, Paisley, Scotland). Bovine serum albumin (BSA) (fraction V), Percoll, HEPES, collagenase type I, human chorionic gonadotropin (hCG), 17β-estradiol and bisphenol A were obtained from Sigma Chemical Co. (St. Louis, MO, USA). PPT and ERB 041 were purchased from Tocris Bioscience (Boston, MA, USA).

### Animals

Inbred one month old males of CBA/Lac, C57BL/6j, BALB/c and 129S2 mouse strains were purchased from Charles River Laboratories International Inc. (Charles-River, Germany) and fed with standard laboratory pellet diet and water *ad libitum*.

### Ethics Statement

Animal studies were approved by the local animal ethics committee (Stockholm North Animal Ethics Committee, permits number Dnr N311/12).

### Isolation and Primary Culture of Leydig Cells

Leydig cells were prepared from the testes of immature mice as described previously [14]. Briefly, after decapsulation and disruption by collagenase treatment (0,25 mg/ml for 20 min at 37°C), the seminiferous tubules were separated mechanically. The interstitial cells were collected by centrifugation at 300 g for 7 min and washed in HBSS containing 0,1% (w/v) BSA. To obtain purified Leydig cells, this suspension was loaded on the top of a discontinuous Percoll gradient, consisting of layers of 20, 40, 60 and 90% Percoll dissolved in Hank’s balanced salts solution and centrifuged at 800 g for 20 min. Subsequently, the fraction enriched in Leydig cells was centrifuged through a continuous self-generated 60% Percoll gradient at 20000 g for 30 min at 4°C. The purity of these Leydig cell preparations was shown to be 90%, as determined by histochemical staining for 3β-hydroxysteroid dehydrogenase [15]. The cell viability, as assessed by Trypan blue exclusion, was greater than 90%. These purified Leydig cells were resuspended in DMEM-F12 supplemented with 15 mM HEPES (pH 7.4), 1 mg/ml BSA, 365 mg/L glutamine, 100 IU/ml penicillin and 100 µg/ml streptomycin.

For culturing, 100 µl of a suspension containing 1.5×10^5^ cells/ml was plated in 96-well plate (Falcon, Franklin Lakes, NJ, USA) and incubated with PPT, ERB041 (selective agonists of ERα and ERβ, respectively), 17β-estradiol (0.1 µM) and bisphenol A (10 µM) with or without hCG (10 ng/ml) at the time of plating of the cells for 17 hours at 34°C in 5% CO_2_. All compounds were dissolved in DMSO and the final concentration of the solvent in the media did not exceed 0.1%.

### Hormone Measurement

Culture medium samples were stored at −20°C prior to analysis of the concentrations of testosterone (T), 17β-estradiol (E_2_), 5α-androstane-3α, 17β-diol and LH. Serum levels of mouse LH were measured by specific ELISA (ABIN415551) from Antibodies-online (Antibodies-online, Inc, GA, USA) in accordance with manufactureŕs instructions. The concentrations of the sex steroids were quantified employing the Coat-a-Count RIA kit (Diagnostic Products Corp., Los Angeles, CA, USA). Intraassay and interassay coefficients for testosterone were 6.4 and 4%, respectively. The same parameters for 17β-estradiol were 4.3 and 5.5%, respectively. Concentration of 5α-androstane-3α,17β-diol was also determined by RIA using specific antisera (Cosmo Bio Co. LTD., Tokyo, Japan). 5α-[9,11-^3^H(N)]Androstane-3α,17β-diol (specificity activity, 40 Ci/mmol) was obtained from NEN Life Science Products (Boston, MA, USA).

### Isolating RNA and Producing cDNA

Total RNA was extracted from control and treatment groups by RNeasy Mini Kit (74104,Qiagen, Hilden, Germany), according to the protocol provided by the manufacturer. The RNA was pretreated with DNAse (RNase-free DNase Set, Qiagen) according to the manufacturer’s instructions. The amount of total RNA was measured by photometry (BioPhotometer, Hamburg, Germany). The RNA was kept at −80°C until analysis. Total RNA was further processed using iScript cDNA Synthesis Kit (Bio-Rad Laboratories, Hercules, CA, USA) as proposed in the manufacturer’s protocol.

### Gene Expression Analysis by qPCR

The samples for qPCR were prepared using iQ SYBR Green Supermix (170–8882, Bio-Rad Laboratories, Hercules, CA, USA) and the PCR cycles were run at 95°C for 10s, 60–62°C for 45s, 95°C for 60s and 55°C for 60s followed by a melting curve from 55–95°C in steps of 0.5°C and then held at 4°C (iCycler iQ, Bio-Rad Laboratories, Hercules, CA, USA) after having estimated the best reaction conditions by running a temperature gradient. All values were normalized to β-actin or Tuba3a as a house keeping gene to balance possible irregularities in RNA concentration. To control effectivity of the process, negative control (RT-) was always added to each qPCR assay. The 2^−ΔΔCt^ method was used to calculate the fold changes in gene expression.

An overview of the used primers and the running conditions are described in Table 1.

**Table 1:** qPCR primer sequences and running conditions, bp-base pair.

Oligo	Sequence	Prod. Length (bp)	Temp (°C)
Actb	F: 5′-acaacggctccggcatgtgcaaag-3′R: 5′-tcccaccatcacaccctggtgccta-3′	107	55–65
Cyp11a1	F: 5′-ggggcaacaagctgcccttcaa-3′R: 5′-tgcagggtcatggaggtcgtgt-3′	88	60
Cyp17a1	F: 5′-ttcgcctgggtaccacaactgc-3′R: 5′-tagagtcaccatctggggccga-3′	110	60
Tuba3a	F: 5′-accagaggcggttgaggacca-3′R: 5′-actcacgcatgctgaactccg-3′	81	55–65
SF-1	F: 5′-ttctgagagcccgctagccact-3′R: 5′-cgtccgctgaacggaaggagaa-3′	116	60
Cyp19	F: 5′-tcggcatgcatgagaacggca-3′R: 5′-cagggcccgtcagagctttcat-3′	93	57
ERα	F: 5′-gtgccaggctttggggacttga-3′R: 5′-atggagcgccagacgagaccaa-3′	98	57
ERβ	F: 5′-tggctgacaaggaactggtg-3′R: 5′-tgaggacctgtccagaacgag-3′	197	64
Sult1e1	F: 5′-gccaaagatgtcgccgtttc-3′R: 5′-aaccatacggaacttgccct-3′	118	63
17βHSD3	F: 5′-aagtgcatgaggttctcgca-3′R: 5′-gtccatgtctggccaactca-3′	151	64
LHR	F: 5′-ctggtgctgaagcagtcaca-3′R: 5′-aggtgagagatagtcgggcg-3′	136	63
5αRI	F: 5′-gatggtgggctcttcctacg-3′R: 5′-aaaaccagcgtcctttgcac-3′	210	63
Gpr30	F: 5′-cggcacagatcaggacaccc-3′R: 5′-tgggtgcatggcagaaatga-3′	120	64
3βHSDI	F: 5′-gcggctgctgcacaggaata-3′R: 5′-gacgcatgcctgcttcgtga-3′	99	63
StAR	F: 5′-aaagccagcaggagaacgggga-3′R: 5′-gcctccatgcggtccacaagtt-3′	133	60

### Statistical Analysis

The differences between various values were analysed for statistical significance by Student’s t-test for pairwise comparison, and the one-way analysis of variance (ANOVA) for multi comparison followed by Holm-Sidak analysis or Dunnett´s analysis if the normality test failed using the SigmaStat (v 11.00) package (SPSS, Inc, Chicago, IL, USA). P<0.05 was considered to be statistically significant.

## Results

### Genotype-dependent Differences in Sex Hormone Levels in Different Mouse Genotypes

**Figure 1:**
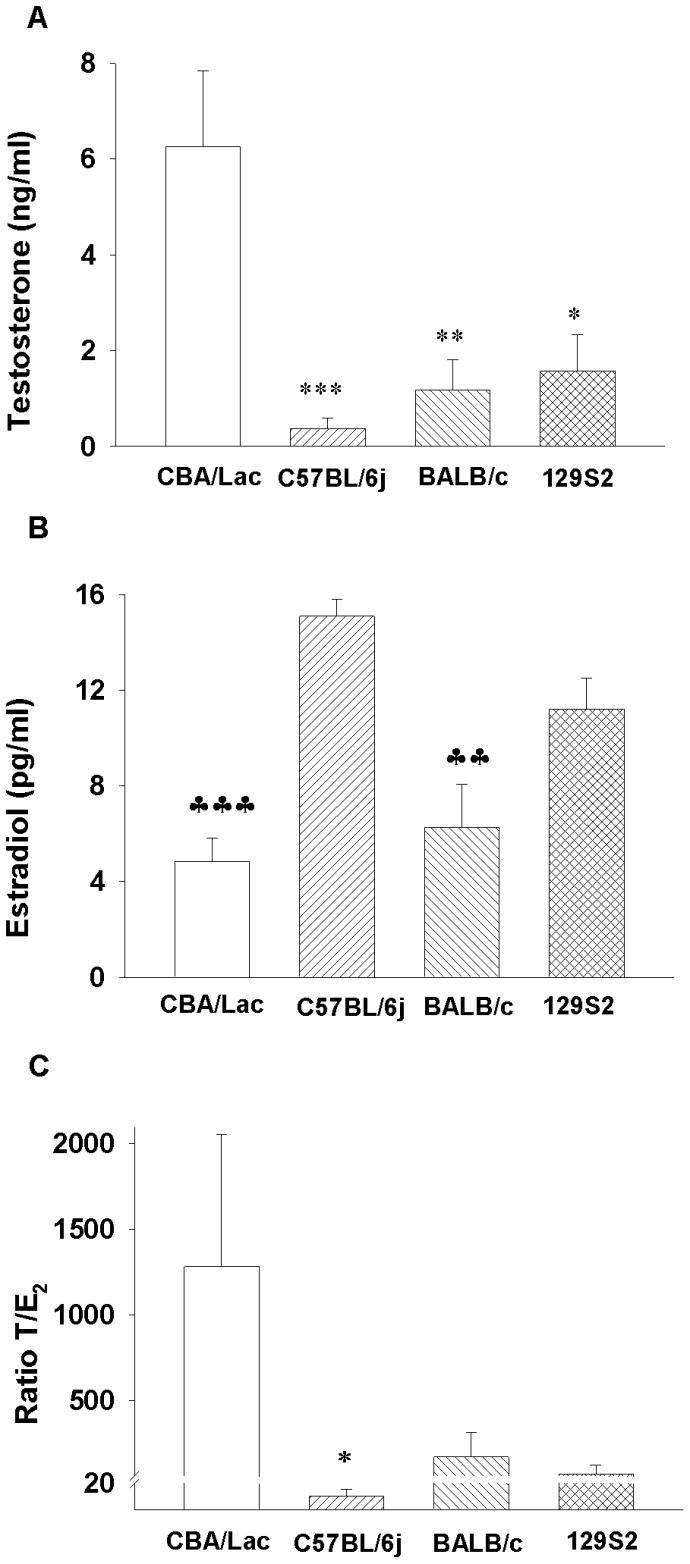
Strain-related variations in sex hormone levels and their ratio in different mouse genotypes. The data are expressed as means ± S.E.M (n = 7–15). *P<0.05, **P<0.01, ***P<0.001compared to CBA/Lac; ♣♣P<0.01, ♣♣♣P<0.01 compared to C57BL/6j.

We first explored genotype-dependent differences in the serum levels of sex hormones in different male mouse genotypes. We observed significant variations in the serum levels of T and E_2_ in different mouse genotypes, where C57BL/6j mice had 20-times lower level of T but 3-times higher serum concentration of E_2_ than CBA/Lac mice (Fig. 1 A, B). Significant variations in sex hormone levels in C57BL/6j and CBA/Lac strains of mice let us to select mouse genotypes with high (CBA/Lac) and low (C57BL/6j) T/E_2_ ratio (Fig. 1C) and suggest significant difference in steroidogenesis in Leydig cells of these mouse genotypes.

### Basal and Human Chorionic Gonadotropin (hCG) Stimulated Androgen and Estrogen Production by Leydig Cells from C57BL/6j and CBA/Lac Mice

**Figure 2:**
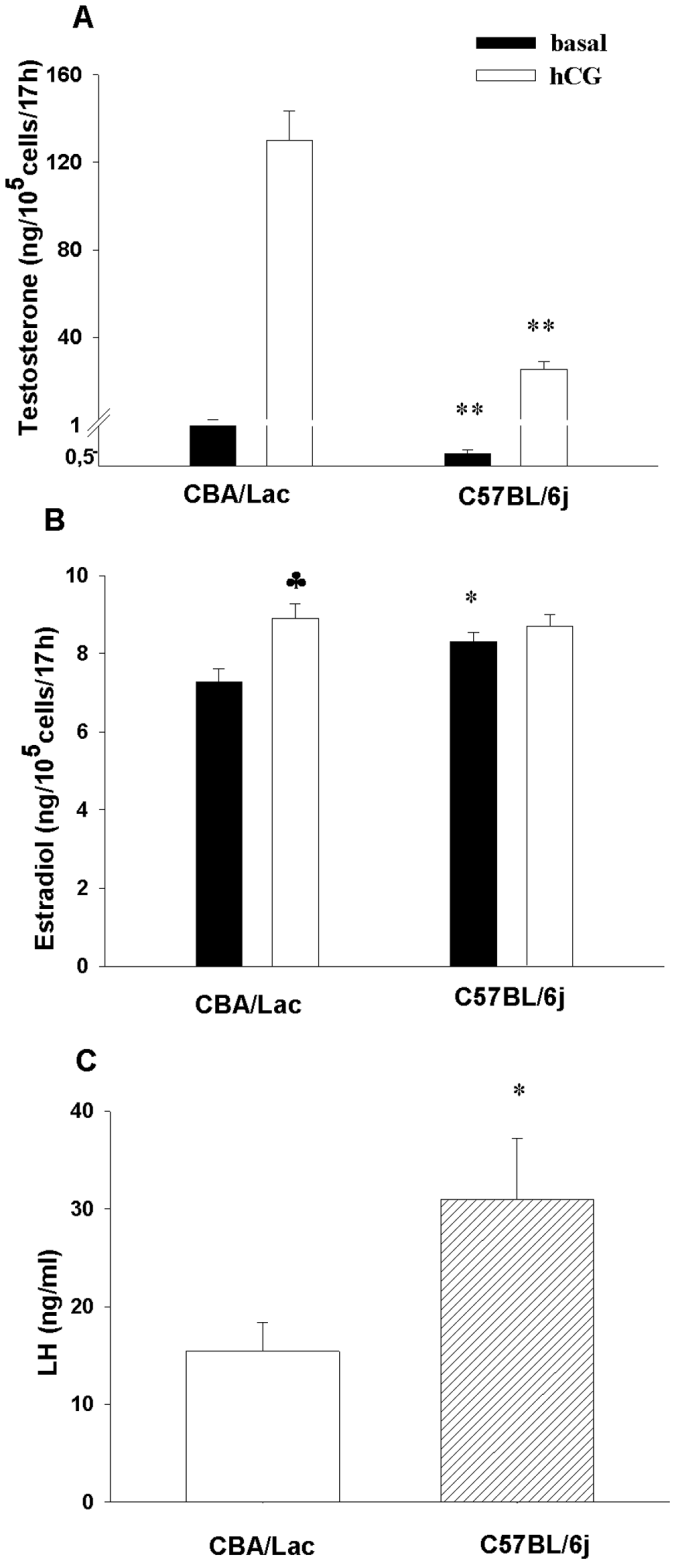
Basal and hCG-stimulated testosterone and estradiol production by Leydig cells from C57BL/6j and CBA/Lac mice as well as serum levels of LH. (A, B) Isolated Leydig cells were incubated with hCG or standard medium (control) for 17h, after which the concentrations of testosterone and estradiol were determined by RIA. Each experiment was performed four times independently obtaining similar results. (C). The data are expressed as means ± S.E.M (n = 23–27). *P<0.05, **P<0.01 compared to CBA/Lac; ♣P<0.05 compared to basal (untreated control).

The previously described low level of T observed in C57BL/6j mice *in vivo* was associated with significantly attenuated capacity of their Leydig cells to produce T in unstimulated basal condition and in response to stimulation with hCG compared to the Leydig cells from CBA/Lac mice (Fig. 2A). In contrast, Leydig cells from C57BL/6j mice were able to synthesize significantly higher levels of E_2_ in basal conditions compared to the Leydig cells from CBA/Lac mice (Fig. 2B). However, hCG-activated Leydig cells from CBA/Lac mice produced a markedly higher amount of E_2_ compared to unstimulated control, while such an effect was not observed in the cells from C57BL/6j mice (Fig. 2B).We further hypothesized that decreased responsiveness to hCG by Leydig cells from C57BL/6j mice can be associated with changes in secretion of LH by the pituitary of this mouse genotype. Indeed, serum level of LH was significantly (1.8-fold, P<0.05) higher in C57BL/6j mice compared to CBA/Lac genotype (Fig. 2C), reflecting feedback mechanism of the compensatory activation of LH release by the pituitary in C57BL/6j mouse genotype.

### Comparative Analysis of the Expression of Steroidogenic Genes in Leydig Cells from C57BL/6j and CBA/Lac Mice

**Figure 3:**
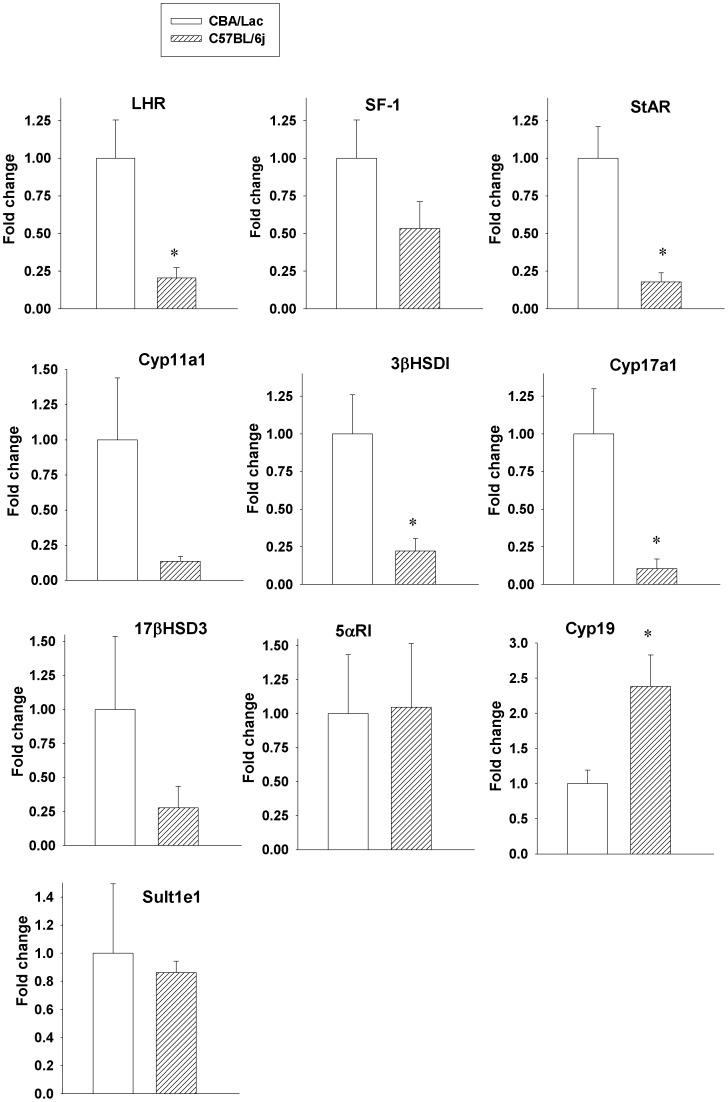
Comparative expression of steroidogenic genes in Leydig cells from CBA/Lac and C57BL/6j mice. Data are expressed as fold change ± S.E.M for four independent RNA preparations. *P<0.05 compared to CBA/Lac.

We further performed comprehensive comparative analysis of the expression of steroidogenic genes to identify molecular basis for attenuated steroidogenesis in Leydig cells from C57BL/6j mice compared to CBA/Lac genotype. We found significant (2.6-fold compared to CBA/Lac, P<0.05) attenuation of the expression of LHR that was associated with coordinated prominent suppression of StAR, 3βHSDI and Cyp17a1 in Leydig cells from C57BL/6j mice compared to CBA/Lac (Fig. 3), a cellular event that may indicate on reduced capacity of LH generates activating signals from LHR to downstream signaling pathways in these cells. Depressed expression of steroidogenic genes involved in the biosynthesis of testosterone was associated with significant (2.4-fold compared to CBA/Lac, P<0.05) activation of Cyp19 expression in Leydig cells from C57BL/6j mice, suggesting that Leydig cells were a major source of serum E_2_ in this genotype.

### Expression of the Estrogen Receptors in Leydig Cells from C57BL/6j and CBA/Lac Mice

**Figure 4:**
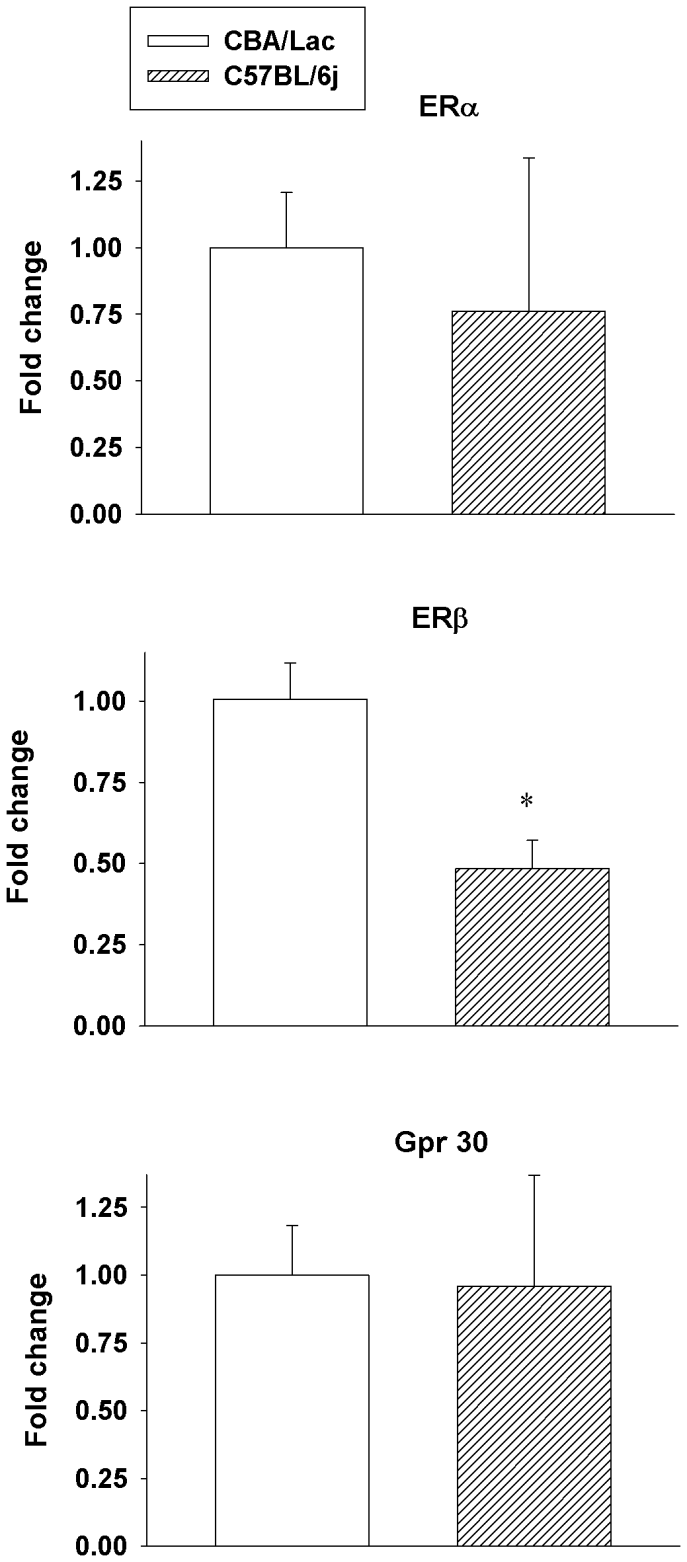
Comparative expression of the family of estrogen receptor genes in Leydig cells from CBA/Lac and C57BL/6j mice. Data are expressed as fold change ± S.E.M for four independent RNA preparations. *P<0.05 compared to CBA/Lac.

We further explored the relationship between androgen production potential of mouse Leydig cells and the levels of the expression of ERs in these cells. We observed significant (2-fold compared to CBA/Lac, P<0.05) reduction in the expression of ERβ in Leydig cells from C57BL/6j mice, while no differences in the expression of ERα and Gpr30 (a transmembrane intracellular estrogen receptor) were found in the androgen-producing cells from both genotypes (Fig. 4).

### Effects of Estrogen Receptor Ligands and Xenoestrogen Bisphenol A on Basal and hCG-stimulated Androgen Production by Leydig Cells from C57BL/6j and CBA/Lac Mice

**Figure 5:**
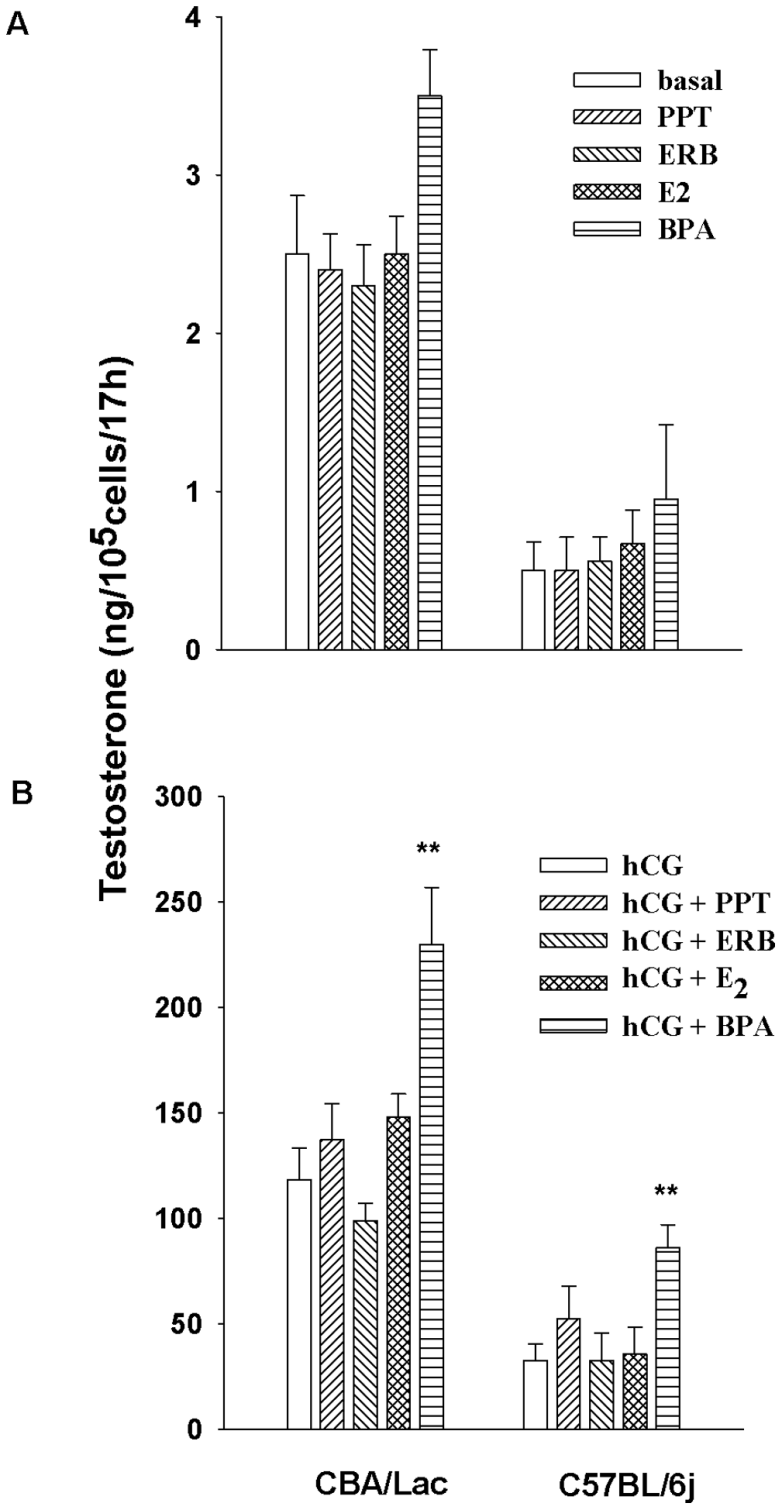
The effect of agonists of estrogen receptors and xenoestrogen BPA on basal and hCG-stimulated testosterone production by Leydig cells from C57BL/6j and CBA/Lac mice. Leydig cells were incubated with the agonists alone (A) or co-treated with hCG for 17 h (B). Each experiment was performed independently five times with similar results. **P<0.01 compared to hCG treatment.

To explore the role of ERs-mediated signaling in the regulation of steroidogenesis in mouse Leydig cells with different capacity to produce androgens, the primary cultures of mouse Leydig cells were treated with selective ERs ligands and xenoestrogen BPA. Our data showed that the activation of ERs with PPT, ERB (selective agonists of ERα and ERβ, respectively) and E_2_ had no significant effect on androgen production by untreated and hCG-stimulated Leydig cells from both mouse genotypes. In contrast, xenoestrogen BPA promoted significantly hCG-activated but not basal androgen production by Leydig cells from C57BL/6j and CBA/Lac mice (Fig. 5).

### Bisphenol A Suppresses Metabolism of Testosterone in Leydig Cells from C57BL/6j and CBA/Lac Mice

**Figure 6:**
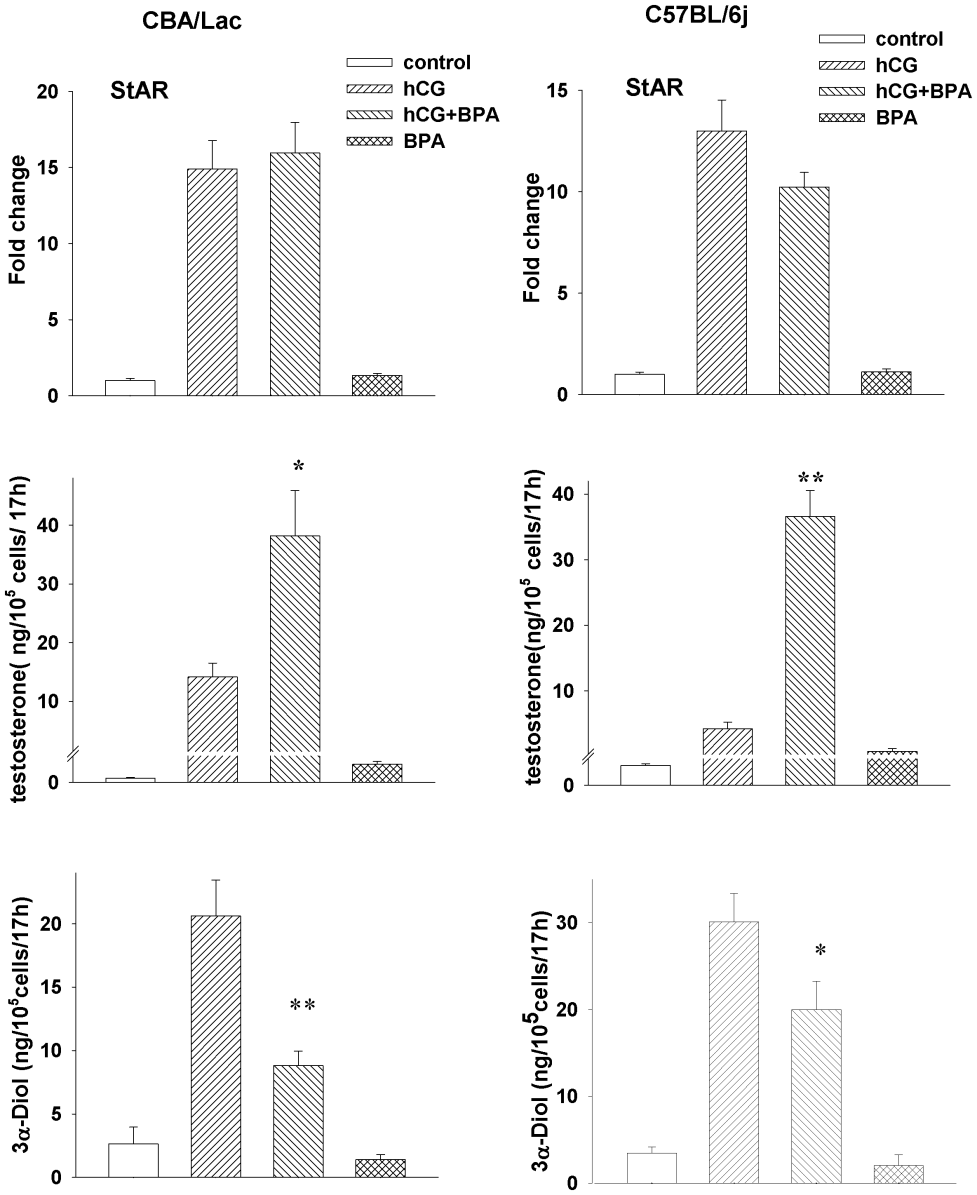
BPA suppresses testosterone metabolism and promotes hCG-stimulated androgen production by mouse Leydig cells. Each experiment was performed independently five times with similar results. Expression of StAR is expressed as fold change ± S.E.M for four independent RNA preparations. *P<0.05, **P<0.01 compared to hCG treatment.

We further investigated mechanism underlying synergistic effect of BPA on hCG-stimulated androgen production by mouse Leydig cells. It was hypothesized that BPA can stimulate biosynthesis of testosterone and/or attenuate its metabolism by immature mouse Leydig cells activated by hCG. Since StAR protein plays pivotal role in initiating and supporting steroidogenesis in Leydig cells [16] and immature rodent Leydig cells can convert testosterone into corresponding steroid metabolite 5α-androstane-3α, 17β-diol [17], we therefore explored modulating effects of BPA on the expression of StAR and the level of 5α-androstane-3α,17β-diol in hCG-stimulated mouse Leydig cells. We found that BPA had no significant effect on hCG-activated StAR expression in Leydig cells from both mouse genotypes but suppressed markedly formation of testosterone metabolite 5α-androstane-3α, 17β-diol resulting in the accumulation of testosterone produced by mouse Leydig cells co-treated with hCG and BPA (Fig. 6).

## Discussion

Since the C57BL/6j mouse genotype could be considered as a naturally occurring mouse model with relatively low androgen but high estrogen levels, we took advantage of this particularity to explore distinctive features of steroidogenesis and sensitivity of their Leydig cells to estrogenic compounds compared to CBA/Lac mice, whose Leydig cells produced relatively high level of testosterone but low concentration of estrogen. The present study is the first to demonstrate that C57BL/6j mouse genotype has relatively low serum level of testosterone and increased serum concentrations of estradiol and LH compared to CBA/Lac mice. These hormonal perturbations were associated with coordinated suppression of the expression of LHR and genes coding steroidogenic enzymes but upregulation of the Cyp19 expression, the cellular events that attenuated steroidogenesis in Leydig cells from C57BL/6j mice. Significant strain-related differences in androgen production by Leydig cells from C57BL/6j and CBA/Lac mice were not tightly linked to variations in the expression of the estrogen receptors ERα and Gpr30, while the expression of ERβ was suppressed in the cells from C57BL/6j and no significant effects of corresponding selective estrogen receptor agonists on basal and hCG-stimulated steroidogenesis in these androgen-producing cells were demonstrated. In contrast to this observation, xenoestrogen BPA significantly promoted hCG-activated androgen production by Leydig cells from C57BL/6j and CBA/Lac mice by attenuating metabolism of testosterone into 5α-androstane-3α, 17β-diol.

Previous studies have shown that C57BL/6j mice have low levels of serum testosterone and were considered as being chronically androgen deficient [18], [19] but mechanisms underlying this phenomenon have not been investigated. Our study has demonstrated that relatively low serum testosterone levels in C57BL/6j mouse genotype were associated with attenuated steroidogenesis in their Leydig cells due to coordinated suppression of the expression of steroidogenic enzyme genes. This specific phenotype of Leydig cells in C57BL/6j mice is thought to be mediated by decreased expression of LHR, a cellular event that can reduce the capacity of LH activate signaling pathways that control steroidogenic gene expression. Therefore, relative androgen deficiency due to low Leydig cell capacity to produce testosterone observed in C57BL/6j can induce the activation of LH secretion by the pituitary via feed-back mechanism to support steroidogenesis in these cells. Further, elevated levels of LH in C57BL/6j mouse genotype can upregulate the expression of Cyp19 observed in our study and increase aromatization of testosterone to estradiol by their Leydig cells. This assumption is supported by the finding that LH has potential to increase Leydig cell capacity to aromatize testosterone to estradiol in vivo and in vitro [20], [21]. Although C57BL/6j male mice have an elevated serum level of estradiol and suppressed androgen production, their spermatogenesis and reproductive potential has been reported to be normal [22], , suggesting that in contrast to humans, low T/E_2_ ratio does not affect the development of germ cells in this mouse genotype. In contrast, genetically modified mice having very high serum levels of estradiol due to the overexpression of the Cyp19 has been demonstrated to have multiple defects in their reproductive organs, disrupted spermatogenesis and suppressed androgen production by Leydig cells [24], an observation that can suggest the presence of threshold of estradiol levels exceeding of which this sex hormone causes negative effects on testicular physiology and fertility.

In the present study we attempt to find out the relationship between androgen production potential of mouse Leydig cells and the levels of the expression of ERs. Our data indicate that there is no clear correlative link between the potency of Leydig cells to produce androgens and the level of ERα expression. We have demonstrated that this subtype of ERs that is coupled with signaling pathways attenuating steroidogenesis in the Leydig cell [4], [6], [7] expressed equally in mouse Leydig cells with different androgen production potential. However, Leydig cells with relatively low potency to produce testosterone from C57BL/6j mice expressed less amount of ERβ, the estrogen receptor that is not involved in the regulation of steroidogenesis in the Leydig cell [7]. The role of ERβ in the regulation of Leydig cell function is as yet weakly investigated. It has been recently reported that the inactivation of the ERβ increased the number of adult Leydig cells per testis that was associated with a decrease in their cell volume, suggesting that the ERβ-coupled signaling pathway(s) may control the number of adult Leydig cells [25]. However, whether the attenuated expression of the ERβ in Leydig cells from C57BL/6j mice might effect on their proliferation in the testis is not yet known. Further, we have shown that direct stimulation of ERs by selective agonists and estradiol did not affect significantly androgen production by developing mouse Leydig cells, suggesting lack of negative effects of ERs-mediated signaling pathways on steroidogenic enzyme genes expression at that stage of the mouse Leydig cell development. This finding is consistent with the studies showing that androgen production by purified immature pig Leydig cells and mouse Leydig cells were not affected by treatment with estradiol [26], [27]. In contrast to immature Leydig cells which are insensitive to direct action of estrogens, androgen production by fetal Leydig cells have been reported to be suppressed by estrogenic compounds in both *in vitro* and *in vivo* experimental paradigms via ERα− mediated signaling [5], [6], [28] but mechanism(s) underlying sensitivity of different populations of Leydig cells to estrogenic stimuli remains to be elucidated.

In contrast to estrogenic agonists, estrogen-like endocrine disruptor BPA significantly potentiated hCG-activated androgen production by Leydig cells from C57BL/6j and CBA/Lac mice but had no effect on basal androgen production. This effect of BPA was associated with suppression of metabolism of testosterone into corresponding steroid metabolite 5α-androstane-3α,17β-diol by immature Leydig cells, the process that reflected attenuated activity of testosterone metabolizing enzymes. One such enzyme, 5α-reductase type I (5αRI), has been shown to play a pivotal role in the biosynthesis of 5α-androstane-3α,17β-diol in the immature mouse testis [29]. However, we did not find significant suppression of the expression of 5αRI by BPA in hCG-treated Leydig cells from both mouse genotypes (data not shown). However, a recent study has reported that BPA can attenuate the expression of 5αRI in the rat prostate after exposure in vivo [30]. This finding allows us to suggest that the suppressive effect of BPA on the expression of 5αRI may be tissue- and species-specific, an assumption that needs further investigation. The observation that BPA has potential to suppress metabolism of testosterone in hCG-activated immature mouse Leydig cells let to suggest that this xenoestrogen may cause premature maturation of Leydig cells and disturb paracrine milieu required for normal development of germ cells.

In our study facilitation of androgen production in hCG-activated Leydig cells by BPA was not associated with the upregulation of the expression of StAR, which initiates and supports steroidogenesis in the Leydig cell [16]. In contrast, BPA has been shown to activate steroidogenesis in mouse Leydig tumor cell line via upregulation of Nur77, an orphan nuclear receptor having potential to activate steroidogenic enzyme gene expression [31]. This discrepancy in the observed effects of BPA on Leydig cell steroidogenesis can be explained by difference in the experimental cell models used in our and above mentioned study (primary culture of native developing mouse Leydig cells *vs* mouse Leydig tumor cells). One can suggest that in contrast to native mouse Leydig cells, androgen-producing tumor cells have altered signaling pathways that facilitate BPA to activate upstream steroidogenic enzyme gene expression and steroidogenesis. Further, in contrast to immature Leydig cells, BPA has been reported to suppress androgen production by fully differentiated rat Leydig cells in vitro [9], indicating on species- and stage-dependent differences in Leydig cell responsiveness to this xenoestrogen. In line with this, recent study has demonstrated species-specific differences in the effects of BPA on androgen production by fetal testes from humans, rats and mice, where human but not rodent fetal testicular steroidogenesis was significantly attenuated by environmentally relevant concentration of BPA [32]. These species-specific differences in response to BPA raised concerns about extrapolation of data from rodent studies to human risk assessment [32].

In summary, the results of the current study show clearly that immature mouse Leydig cells are resistant to estrogenic stimuli and their potency to produce androgens is not tightly linked to the levels of the expression of ERα. In contrast, xenoestrogen BPA facilitates hCG-induced androgen production in immature mouse Leydig cells by attenuating conversion of testosterone into androgen metabolite 5α-androstane-3α,17β-diol. This cellular event can disturb differentiation of the Leydig cell and create abnormal intra-testicular paracrine milieu that may alter proper development of germ cells. Our findings also indicate that C57BL/6j mouse genotype that is widely used in laboratory experiments have relatively low serum levels of testosterone due to attenuated steroidogenesis in their Leydig cells, an observation that needs to take into consideration in experimental paradigms where androgen-dependent processes are investigated.
